# Association between weight-adjusted-waist index and risk of cardiovascular diseases in United States adults: a cross-sectional study

**DOI:** 10.1186/s12872-023-03452-z

**Published:** 2023-09-01

**Authors:** Haiyang Fang, Feng Xie, Kai Li, Meng Li, Yanqing Wu

**Affiliations:** 1 Department of Cardiovascular Medicine, Nanchang University Second Affiliated Hospital, No.1 Minde Road, 330006 Nanchang, Jiangxi P.R. of China; 2 No.1 Minde Road, 330006 Nanchang, Jiangxi P.R. of China

**Keywords:** Weight-adjusted-waist index, Obesity, Cardiovascular disease, NHANES

## Abstract

**Background:**

As a new obesity-related index, the weight-adjusted-waist index (WWI) appears to be a good predictor of cardiovascular disease (CVD) in East Asian populations. This study aimed to validate the association between WWI and CVD in United States (US) adults and also evaluate its relationships with the prevalence of specific CVDs.

**Methods:**

The data were obtained from the 2009–2016 National Health and Nutrition Examination Survey. WWI was calculated as waist circumference divided by the square root of weight, and CVD was ascertained based on self-reported physician diagnoses. Multivariable logistic regression models and subgroup analyses were performed to evaluate the association between WWI and CVD.

**Results:**

A total of 21,040 participants were included. There was a positive linear relationship between WWI and the odds of CVD (*P* = 0.310). After adjusting for all covariates, each unit of increased WWI was associated with 48% increased risk of CVD (odds ratio [OR]: 1.48, 95% confidence interval [CI]: 1.25–1.74). Moreover, compared with the lowest quintile (< 10.3 cm/√kg), the multivariable-adjusted OR was 3.18 (95% CI: 1.80–5.59) in the highest quintile (≥ 11.8 cm/√kg). Besides, positive associations were also found between WWI and increased prevalence of congestive heart failure (OR: 1.47, 95% CI: 1.11–1.96), coronary heart disease (OR: 1.27, 95% CI: 1.01–1.60), angina (OR: 1.44, 95% CI: 1.06–1.96), heart attack (OR: 1.66, 95% CI: 1.29–2.12), and stroke (OR: 1.32, 95% CI: 1.02–1.70). Subgroup analyses showed that stronger associations between WWI and CVD were detected in participants younger than 50 years of age (*P* < 0.001).

**Conclusions:**

High levels of WWI were significantly associated with an increased risk of CVD in US adults, particularly in people under 50 years of age. These findings indicate that WWI may be an intervention indicator to reduce the risk of CVD in the general adult population.

**Supplementary Information:**

The online version contains supplementary material available at 10.1186/s12872-023-03452-z.

## Background

Obesity is a major public health problem worldwide. The global prevalence of obesity has doubled since 1980, and it is expected to reach 18% in men and surpass 21% in women by 2025 [1, 2]. According to statistics from the National Health and Nutrition Examinations Survey (NHANES) 2015–2016, approximately 39.6% of adults and 18.5% of youths were obese in the United States (US) [3]. Furthermore, large-scale and long-term studies have consistently demonstrated that obesity is associated with a significantly increased risk of cardiovascular disease (CVD) morbidity and mortality [4, 5]. Therefore, early identification of obese individuals with high risk is crucial to preventing CVD.

Body mass index (BMI) and waist circumference (WC) are the most commonly used obesity-related indices. Accumulating data has indicated that they are strongly associated with a rise in the prevalence of hypertension, stroke, and other CVDs [6, 7]. However, in recent years, controversy regarding the health outcomes of overweight and obesity has grown in CVD patients, given findings of similar or lower all-cause mortality compared with their normal-weight counterparts [8–10]. This is partly because these anthropometric measures do not clearly distinguish between muscle mass and fat mass [11, 12]. Although the waist-to-height ratio (WHtR) appears to be superior in assessing obesity, it remains controversial in predicting obesity-related CVD risk and mortality [13, 14]. As a result, these traditional indices may not accurately reflect the relationship between obesity and CVD risk.

In this context, a new anthropometric index called the weight-adjusted-waist index (WWI) was proposed, which standardized WC for body weight. Subsequently, Park et al. found that in the Korean National Cohort study, WWI was a better predictor of CVD mortality than BMI, WC, and WHtR. Also, only WWI demonstrated a linear positive correlation between adiposity indices and cardiovascular mortality, but not BMI, WC, or WHtR [15]. Additionally, several prospective studies in China discovered that higher WWI levels were associated with an increased risk of all-cause and cardiovascular mortality [16, 17]. Unfortunately, the associations were only validated in East Asian populations. Moreover, there is relatively little information on the relationships between WWI and the prevalence of specific CVDs (such as coronary heart disease and stroke).

To address the knowledge gap, this study aimed to investigate the associations of WWI, an innovative anthropometric index, with CVD and its subtypes in US adults based on data obtained from the NHANES.

## Methods

### Study design

NHANES is an ongoing cross-sectional survey administered by the National Center for Health Statistics (NCHS), which is part of the Centers for Disease Control and Prevention (CDC), to assess the health and nutritional status of adults and children in the United States. It documents a repeated two-year cycle with five major parts, including demographic data, dietary data, examination data, laboratory data, and questionnaire data. Due to the multi-stage, stratified, and probability sampling design, the included participants showed relatively great representativeness. The details of NHANES study design and methods have been previously described [18]. The NHANES study protocol was approved by the NCHS Research Ethics Review Board, and written informed consent was obtained from each participant.

### Study population

We used the continuous NHANES data from 2009 to 2016 (N = 40,439). Participants younger than 18 years of age (N = 15,943) and those with incomplete CVD evaluation data (N = 1,231) were excluded. Then, we further excluded participants with missing data on weight and WC value (N = 2,225). Finally, a total of 21,040 participants were included for current analysis. The detailed flow chart of participant selection is shown in Additional File Fig. 1. All data included in this manuscript are publicly available at https://www.cdc.gov/nchs/nhanes/.

### Covariates of interest

Potential covariates, including demographic data (age, gender, educational level, and race/ethnicity), lifestyle variables (smoking status and alcohol drinking), anthropometric measurements (height, weight, WC, and blood pressure [BP]), and laboratory results (hemoglobin A1c [HbA1c], total bilirubin [TBIL], low-density lipoprotein cholesterol [LDL-C], high-density lipoprotein cholesterol [HDL-C], total cholesterol [TC], triglycerides [TG], serum creatinine [Scr], serum uric acid [SUA], and urinary albumin/creatinine ratio [UACR]) were selected based on clinical relevance and statistical significance. Demographics and lifestyle data were derived from the household interview questionnaires administered by highly trained medical personnel. Anthropometric indicators and biochemical parameters were obtained through medical examinations and subsequent laboratory assessments in the Mobile Examination Centre (MEC).

The educational level was further categorized as less than 9th grade, 9-11th grade, high school graduate, some college or AA degree, and college graduate or above. Race/ethnicity was classified as Mexican American, other Hispanic, non-Hispanic black, non-Hispanic white, and Other Race (including multi-racial). The smoking status was determined by “Smoked at least 100 cigarettes in life”, and the alcohol drinking status was evaluated by “Had at least 12 alcohol drinks per year”. The history of asthma and cancer was examined through the item “Ever been told you have asthma and/or cancer or malignancy”. The laboratory results, including HbA1c, TBIL, LDL-C, HDL-C, TC, TG, Scr, SUA and UACR levels were determined using standardized methods. The detailed measurement processes of these variables are publicly available at https://www.cdc.gov/nchs/nhanes/. Additionally, the BMI in kg/m^2^ was calculated by dividing weight (kg) by the square of height (m^2^), and the WHtR was determined by WC (cm) divided by height (cm). The estimated glomerular filtration rate (eGFR) was calculated using the recently published Chronic Kidney Disease Epidemiology Collaboration creatinine equation [19].

### Exposure variable and outcomes

In this study, WWI (cm/√kg) was designed as exposure variable. WWI was developed according to the formula ^*[In (WC) = β0 + β1 In (weight) + ε]*^. Since the estimated β_1_ was 0.494 (close to 0.5), the final formula of WWI was calculated as WC (cm) divided by the square root of weight (kg) [15]. Weight was measured to the nearest 0.1 kg using a digital weight scale, and WC was measured by a retractable steel measuring tape, positioning the measuring tape around the waist at the uppermost lateral border of the ilium at the midaxillary line. The full procedure, including the protocols, equipment, and quality control, was described at https://wwwn.cdc.gov/nchs/nhanes/continuousnhanes/manuals.aspx?BeginYear=2009.

The outcome variable was CVD. According to previous studies, CVD was defined as a composite of 5 self-reported cardiovascular outcomes, which included congestive heart failure (CHF), coronary heart disease (CHD), angina/angina pectoris, heart attack, and stroke [20]. All participants were asked the following questions: “Has a doctor or other health professional ever told you that you have congestive heart failure/coronary heart disease/angina pectoris/heart attack/stroke? ” Participants who answered “yes” to any of the questions were considered to have CVD. Additionally, we also collected data for each CVD subtype to further analyze the association with WWI.

### Statistical analysis

All statistical analyses were performed in accordance with NHANES analytic guidelines [21]. Four waves of continuous survey data (NHANES 2009–2010, 2011–2012, 2013–2014, and 2015–2016) were combined, and an 8-year sampling weight was calculated by using a quarter of the 2-year sampling weight (WTMEC2YR).

Data are presented as weighted mean ± standard deviation (SD) or median (interquartile range, IQR) for continuous variables, and frequency (weighted percentage) for categorical variables. Comparisons between the CVD and non-CVD groups were performed using either the weighted Chi-square test (categorical variables) or the weighted linear regression model (continuous variables). In sensitivity analysis, WWI was converted from a continuous variable to a categorical variable (quintiles) to evaluate its robustness. Multivariable logistic regression model was used to calculate odds ratios (ORs) and corresponding 95% confidence intervals (CIs) to determine the prevalence of CVD related to WWI. We developed three models to adjust for potential confounders: model 1 was a crude model; model 2 was adjusted for age, gender, and race; model 3 was the same as model 2 with additional adjustment for education level, smoking, alcohol drinking, eGFR, systolic and diastolic BP, HbA1c, TBIL, LDL-C, HDL-C, TC, TG, Scr, SUA and UACR. The restricted cubic spline model was used for the dose-response analysis between WWI and total CVD. Subgroup analysis stratified by gender, age, race, BMI (< 25 / 25–30 / ≥ 30 kg/m^2^), WC (abnormal: WC ≥ 94 cm in male and WC ≥ 80 cm in female), eGFR (< 90 / ≥ 90 ml/min/1.73m^2^), smoking, and alcohol drinking was conducted by stratified multivariate regression analysis. A receiver operating characteristic (ROC) curve was used to analyze the predictive value of WWI and traditional obesity-related indices (BMI, WC, and WHtR) for CVD. Besides, we used multiple imputation (MI), based on 5 replications and the Markov-chain Monte Carlo method in the SAS MI procedure, to account for missing data on BMI, systolic BP, diastolic BP, alcohol consumption, TG, and LDL-C (Model 3).

All analyses were performed using R version 4.0.3 (www.R-project.org) and EmpowerStates (www.empowerstats.com). A two-sided *P* value of < 0.05 was considered statistically significant.

## Results

### Baseline characteristics

The characteristics of the study population are presented in Table 1. A total of 21,040 participants were included in this study, 51.47% of whom were females, with an average age of 47.11 ± 16.79 years. The weighted mean WWI was 10.98 ± 0.83 cm/√kg overall, and the weighted prevalence of total CVD, CHF, CHD, angina, heart attack, and stroke were 7.91% (N = 2063), 2.20% (N = 599), 3.21% (N = 794), 1.96% (N = 481), 3.12% (N = 810) and 2.47% (N = 691), respectively. Compared with the non-CVD group, the CVD group was older and more likely to be male; to have higher BMI, WC, WHtR, WWI, SBP, HbA1c, TG, Scr, SUA, and UACR levels; to have a higher proportion of smoking and non-Hispanic White individuals; to have a lower rate of alcohol drinking; and to have lower eGFR, LDL-C, and HDL-C levels (all *P* < 0.05).

**Table 1:** Baseline characteristics of the study participants

Variables^#^	Total (n = 21,040)	Non-CVD (n = 18,977)	CVD (n = 2063)	*P* value
Age (years)	47.11 ± 16.79	45.65 ± 16.25	64.15 ± 13.32	< 0.001
**Gender (%)**				< 0.001
Male	10,243 (48.53)	9059 (47.93)	1184 (55.46)	
Female	10,797 (51.47)	9918 (52.07)	879 (44.54)	
**Race (%)**				< 0.001
Mexican American	3153 (8.65)	2941 (8.96)	212 (5.04)	
Other Hispanic	2263 (5.97)	2078 (6.14)	185 (4.08)	
Non-Hispanic White	8505 (66.26)	7449 (65.73)	1056 (72.50)	
Non-Hispanic Black	4424 (11.12)	3973 (11.06)	451 (11.70)	
Other Race	2695 (8.01)	2536 (8.12)	159 (6.68)	
**Educational level (%)**				< 0.001
< 9th grade	2135 (5.45)	1832 (5.16)	303 (8.83)	
9−11th grade	2915 (10.56)	2551 (10.22)	364 (14.57)	
High school	4630 (21.28)	4114 (20.86)	516 (26.18)	
College	6273 (32.09)	5715 (32.18)	558 (30.98)	
Graduate or above	5070 (30.57)	4750 (31.53)	320 (19.41)	
BMI (kg/m^2^)	28.94 ± 6.74	28.79 ± 6.68	30.65 ± 7.25	< 0.001
WC (cm)	99.20 ± 16.38	98.57 ± 16.21	106.57 ± 16.57	< 0.001
WHtR	0.59 ± 0.10	0.59 ± 0.10	0.64 ± 0.10	< 0.001
WWI (cm/√kg)	10.98 ± 0.83	10.93 ± 0.82	11.55 ± 0.75	< 0.001
Smoking (%)	9183 (44.00)	7933 (42.46)	1250 (61.91)	< 0.001
Alcohol drinking (%)	13,935 (77.98)	12,579 (78.38)	1356 (73.57)	< 0.001
SBP (mmHg)	122.11 ± 17.17	121.49 ± 16.75	129.32 ± 20.04	< 0.001
DBP (mmHg)	70.52 ± 12.17	70.76 ± 11.96	67.66 ± 14.11	< 0.001
eGFR (ml/min/1.73m^2^)	118.05 ± 46.03	120.31 ± 45.50	91.71 ± 43.90	< 0.001
HbA1c (%)	5.63 ± 0.93	5.59 ± 0.88	6.17 ± 1.25	< 0.001
TBIL (umol/L)	11.49 ± 5.24	11.48 ± 5.22	11.71 ± 5.44	0.082
HDL-C (mmol/L)	1.39 ± 0.44	1.39 ± 0.44	1.29 ± 0.43	< 0.001
LDL-C (mmol/L)	2.95 ± 0.91	2.99 ± 0.90	2.55 ± 0.88	< 0.001
TC (mmol/L)	5.01 ± 1.08	5.04 ± 1.07	4.63 ± 1.09	< 0.001
TG (mmol/L)	1.40 ± 1.18	1.38 ± 1.17	1.57 ± 1.19	< 0.001
Scr (umol/L)	77.91 ± 30.25	76.56 ± 27.31	93.57 ± 51.11	< 0.001
SUA (umol/L)	321.45 ± 83.23	318.95 ± 82.03	350.49 ± 91.20	< 0.001
UACR (mg/g)	7.05 (4.55, 13.33)	6.78 (4.46, 12.22)	11.82 (6.16, 33.19)	< 0.001
Asthma (%)	3063 (15.04)	2630 (14.46)	433 (21.85)	< 0.001
Cancer or malignancy (%)	1945 (10.24)	1505 (9.14)	440 (23.09)	< 0.001

^#^ Values are presented as weighted mean ± standard deviation, medians (interquartile range), or frequency (weighted percentages) when appropriate. Abbreviations: CVD, cardiovascular disease; BMI, body mass index; WC, waist circumference; WHtR, waist-to-height ratio; WWI, weight-adjusted-waist index; SBP, systolic blood pressure; DBP, diastolic blood pressure; eGFR, estimated glomerular filtration rate; HbA1c, hemoglobin A1c; TBIL, total bilirubin; HDL-C, high-density lipoprotein cholesterol; LDL-C, low-density lipoprotein cholesterol; TC, total cholesterol; TG, triglycerides; Scr, serum creatinine; SUA, serum uric acid; UACR, urinary albumin/creatinine ratio.

### Association of WWI with CVD

**Fig. 1:**
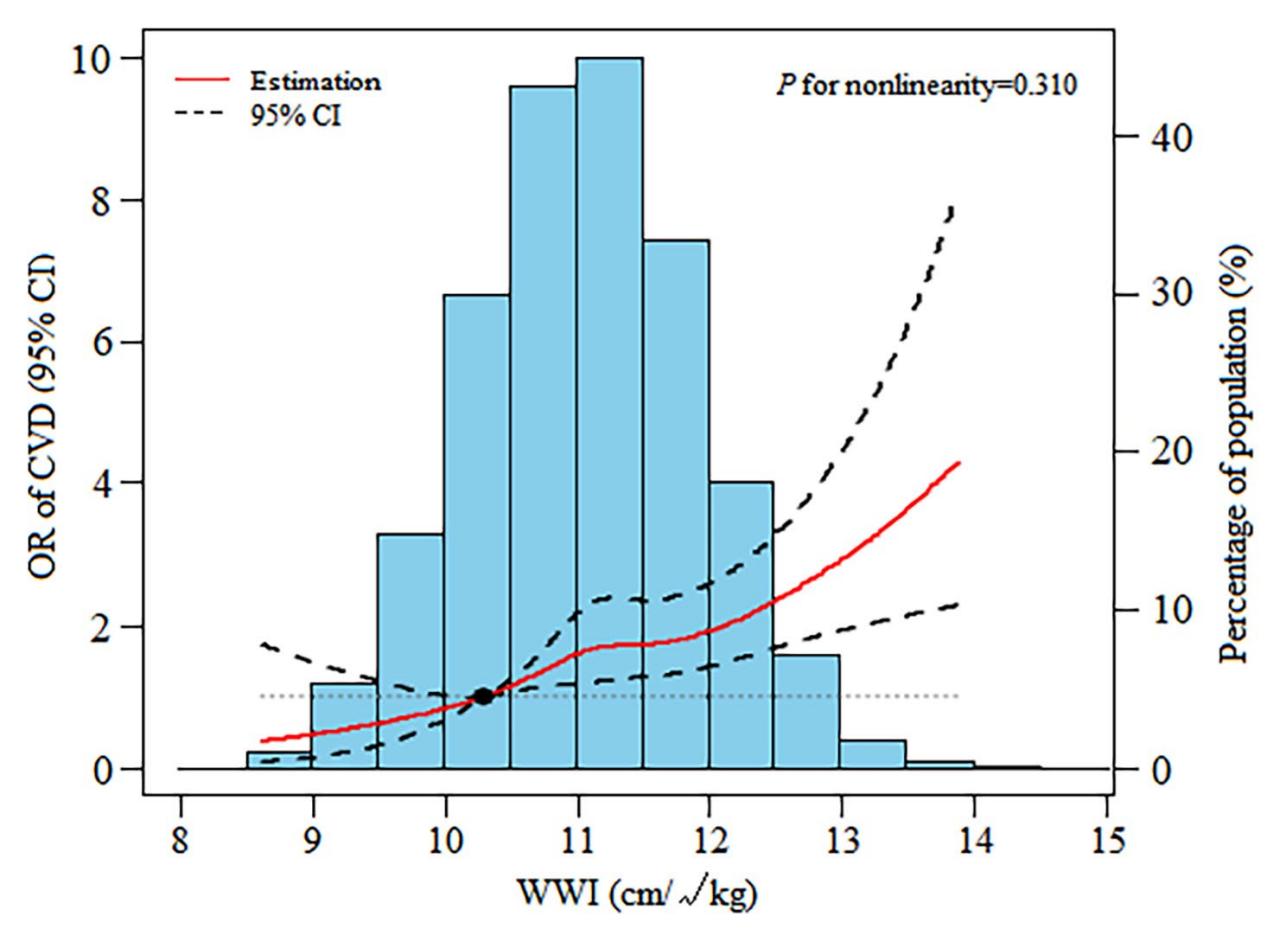
Dose-response relationship between WWI and the risk of CVD. Adjusted odds ratio of total CVD from a restricted cubic spline logistic regression model with knots at the 5th, 35th, 65th, and 95th percentiles. Data are ORs (solid line) and 95% CIs (dashed lines) from multivariate logistic regression analysis with restricted cubic splines. Abbreviations: WWI, weight-adjusted-waist index; CVD, cardiovascular disease; OR, odds ratio; CI, confidence interval

Multivariable logistic regression models were performed to explore the association between CVD and the WWI as continuous and categorical variables (Table 2). When WWI was analyzed as a continuous variable, we found that increased WWI was associated with a higher risk of CVD (Model 1: OR = 2.50, 95% CI: 2.33–2.69; Model 2: OR = 1.74, 95% CI: 1.58–1.92; all *P* < 0.001). In the fully adjusted Model 3, the results indicated that each unit of increased WWI was associated with 48% increased risk of CVD (OR = 1.48, 95% CI: 1.25–1.74, *P* < 0.001). In sensitivity analysis, the multivariable-adjusted ORs (reference to Quintile 1) was 1.74 (95% CI: 1.00-3.02; *P* = 0.048) for Quintile 2, 2.38 (95% CI: 1.38–4.13; *P* = 0.002) for Quintile 3, 2.56 (95% CI: 1.47–4.45; *P* < 0.001) for Quintile 4, and 3.18 (95% CI: 1.80–5.59; *P* < 0.001) for Quintile 5, indicating a stable positive association between higher WWI and increased risk of CVD (*P* for trend < 0.001). Moreover, we reanalyzed the association between WWI and CVD using imputation data and the results did not change qualitatively (Additional File Table 1). In addition, the restricted cubic spline with a multivariate logistic regression model revealed that there was a positive linear relationship between WWI and the odds of CVD (*P* for nonlinear = 0.310) (Fig. 1).

**Table 2:** Adjusted odds ratios (95% CI) for association between WWI and the prevalence of CVD.

WWI (cm/√kg)	Events (%)	OR (95% CI), *P* value		
		Model 1	Model 2	Model 3
Per 1 cm/√kg increase	2063 (7.91)	2.50 (2.33, 2.69) < 0.001	1.74 (1.58, 1.92) < 0.001	1.48 (1.25, 1.74) < 0.001
Categorical				
Quintile 1 (< 10.3)	108 (1.75)	Reference	Reference	Reference
Quintile 2 (10.3–10.8)	215 (3.95)	2.31 (1.69, 3.14) < 0.001	1.42 (1.04, 1.96) 0.027	1.74 (1.00, 3.02) 0.048
Quintile 3 (10.8–11.3)	391 (7.96)	4.85 (3.64, 6.45) < 0.001	2.25 (1.67, 3.04) < 0.001	2.38 (1.38, 4.13) 0.002
Quintile 4 (11.3–11.8)	560 (11.19)	7.06 (5.36, 9.30) < 0.001	2.65 (1.96, 3.58) < 0.001	2.56 (1.47, 4.45) < 0.001
Quintile 5 (≥ 11.8)	789 (17.57)	11.94 (9.12, 15.63) < 0.001	3.87 (2.86, 5.22) < 0.001	3.18 (1.80, 5.59) < 0.001
*P* for trend		< 0.001	< 0.001	< 0.001

Model 1: crude model;

Model 2: adjusted for age, gender and race;

Model 3: adjusted for age, gender, race, education level, smoking, alcohol drinking, systolic blood pressure, diastolic blood pressure, estimated glomerular filtration rate, hemoglobin A1c, total bilirubin, high-density lipoprotein cholesterol, low-density lipoprotein cholesterol, total cholesterol, triglycerides, serum creatinine, serum uric acid, and urinary albumin/creatinine ratio. The covariates were determined based on the matched odds ratio changed at least 10% when added to this model. Abbreviations: WWI, weight-adjusted-waist index; CVD, cardiovascular disease; OR, odds ratio; CI, confidence interval.

### Association between WWI and specific CVDs

We further analyzed the associations between WWI and the prevalence of five specific CVDs (CHF, CHD, angina, heart attack, and stroke) (Additional File Table 2). After adjusting for all covariates (Model 3), the fifth quintile of WWI was positively associated with the increased prevalence of CHF (quintile 5: OR = 2.67, 95% CI: 1.12–6.33, *P* = 0.026), angina (quintile 5: OR = 3.16, 95% CI: 1.05–9.54, *P* = 0.041) and heart attack (quintile 5: OR = 13.68, 95% CI: 4.93–38.03, *P* < 0.001), and the fourth quintile of WWI was associated with the increased prevalence of CHD (quintile 4: OR = 2.57, 95% CI: 1.06–6.27, *P* = 0.037).

### Subgroup analysis

**Fig. 2:**
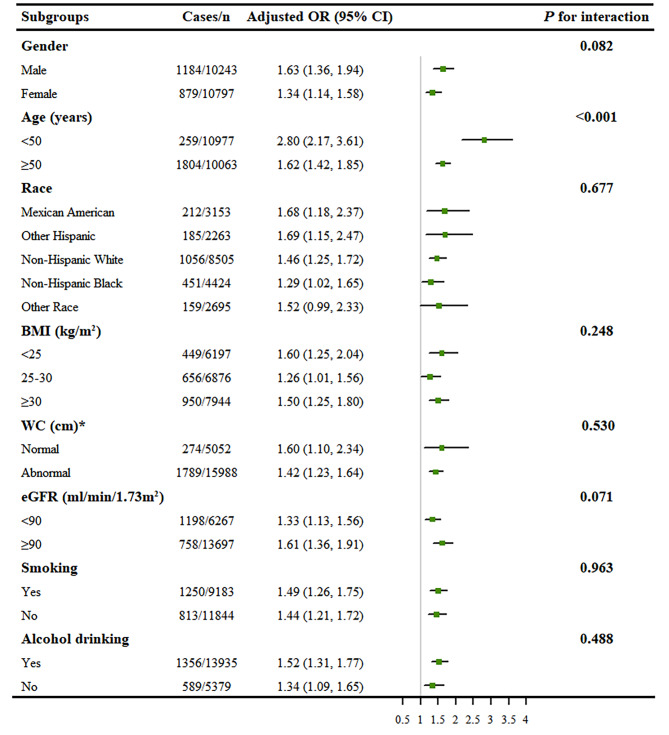
Subgroup analyses of the association between WWI and CVD in US adults. All presented covariates were adjusted (as Model 3) except the corresponding stratification variable. ^*^ Normal: WC < 94 cm in male and WC < 80 cm in female; abnormal: WC ≥ 94 cm in male and WC ≥ 80 cm in female. Abbreviations: WWI, weight-adjusted-waist index; CVD, cardiovascular disease; US, the United States; BMI, body mass index; WC, waist circumference; eGFR, estimated glomerular filtration rate; OR, odds ratio; CI, confidence interval

We performed a further stratified analysis to assess the effect of WWI on CVD in various subgroups (Fig. 2). None of the variables, including gender (female or male), race (Mexican American, other Hispanic, non-Hispanic white, non-Hispanic black and other race), BMI (< 25 or 25–30 or ≥ 30 kg/m^2^), WC (normal or abnormal), eGFR (< 90 or ≥ 90 ml/min/1.73m^2^), smoking (yes or no), and alcohol drinking (yes or no) significantly modified the association between WWI and CVD (all *P* for interaction > 0.05). Nevertheless, there was a significant interaction between WWI and age (< 50 or ≥ 50 years) on CVD. A stronger positive association between WWI and CVD was found in participants younger than 50 years of age (OR = 2.80, 95% CI: 2.17–3.61) compared with their counterparts (OR = 1.62; 95% CI: 1.42–1.85) (*P* for interaction < 0.001).

### ROC curves between WWI and traditional obesity indices

The three traditional obesity indices (BMI, WC, and WHtR) were significantly correlated with one another, with Pearson correlation coefficients that ranged from r = 0.908 to 0.930; however, there was a fair correlation between WWI and those indices (> 0.5 r < 0.8) (Additional File Table 3). The ROC curves of the different indices for CVD are shown in Table 3. For both males and females, WWI had the highest area under the curve (AUC) of 0.736 (95% CI: 0.722–0.751, *P* < 0.05) and 0.677 (95% CI: 0.659–0.694, *P* < 0.05), with a cut-off value of 11.11 cm/√kg and 11.47 cm/√kg, respectively.

**Table 3:** Comparison of the ability of different obesity-related indices to predict CVD.

Test	AUC (95% CI)	Cut-off	Specificity	Sensitivity	Youden index	*P* value
Males						
BMI	0.567 (0.549, 0.584)	27.86	0.523	0.579	0.102	< 0.001
WC	0.638 (0.622, 0.653)	97.15	0.463	0.752	0.215	< 0.001
WHtR	0.659 (0.643, 0.674)	0.58	0.542	0.705	0.247	< 0.001
WWI	0.736 (0.722, 0.751)	11.11	0.654	0.709	0.363	< 0.001
**Females**						
BMI	0.569 (0.550, 0.589)	29.62	0.579	0.533	0.112	< 0.001
WC	0.612 (0.593, 0.631)	104.45	0.699	0.464	0.163	< 0.001
WHtR	0.629 (0.610, 0.647)	0.59	0.504	0.694	0.198	< 0.001
WWI	0.677 (0.659, 0.694)	11.47	0.619	0.639	0.258	< 0.001

Abbreviations: WWI, weight-adjusted-waist index; BMI, body mass index; WHtR, waist-to-height ratio; WC, waist circumference; AUC, area under the curve; CI, confidence interval

## Discussion

To our knowledge, this cross-sectional study was the first to demonstrate the relationship between WWI and CVD in a nationally representative sample of the US adult population. The results indicated that WWI was significantly associated with an increased risk of CVD, presenting a nearly linear dose-response relationship. Furthermore, the subgroup analyses showed that a stronger association between WWI and CVD was detected in participants younger than 50 years of age (*P* for interaction < 0.001). Besides, the ROC curve showed that WWI had better identification performance for CVD than BMI, WC, and WHtR.

Obesity, a condition of excessive body fat accumulation caused by long-term energy intake exceeding energy expenditure, is a well-established risk factor for CVD and mortality in adult populations [5]. Hence, measurement of body fat has been a critical issue in clinical practice for assessing obesity and then identifying individuals at risk of CVD. Although cumulative evidence shows that those traditional obesity-related indices (such as BMI, WC, and waist-to-hip ratio [WHR]) are associated with CVD risk, the obesity paradox still exists, partly because the significant correlations between various indicators hamper the identification of biologically driven risks for diseases (as shown in Table S3) [7, 22, 23]. Another explanation for the paradox may be that BMI cannot distinguish between an elevated weight caused by high levels of lean vs. fat body mass. In this context, nontraditional obesity indicators (such as body shape index [ABSI], cardiometabolic index [CMI], and visceral adiposity index [VAI]) were also employed to assess the exact relationship between them and find positive associations [24–26]. For example, a prospective study of 3,042 participants found that VAI was significantly associated with the 10-year CVD incidence (HR = 1.05, 95% CI: 1.01–1.10) [26]. Some scholars also put forward “metabolic health” based on WHR, systolic BP, and self-reported diabetes and found it to be clearly associated with CVD and total mortality, regardless of BMI [27]. However, those parameters are complex to calculate or susceptible to subjectivity, making it inconvenient to conduct routine examinations in the general population.

WWI is a simple anthropometric indicator derivated according to the formula ^*[In (WC) = β0 + β1 In (weight) + ε]*^, which standardizes WC for weight by the least squared regression of the logarithm-transformed WC on the logarithm-transformed weight [15]. Hence, WWI may combine the advantages of WC while weakening the correlation with BMI, enabling it to evaluate both fat mass and muscle mass. Indeed, a recent study involving 602 participants showed that WWI was positively correlated with total and abdominal fat measures but negatively correlated with appendicular skeletal muscle mass in older adults [28]. Moreover, WWI was significantly higher in the metabolically unhealthy group than in the metabolically healthy group, even with similar levels of obesity [29]. Thus, WWI can reflect “true obesity” that is metabolically unhealthy. Several studies have demonstrated that WWI is positively correlated with CVD mortality in East Asian populations [15–17]. Our study further verified the association between WWI and CVD in the US adult population. Furthermore, the WWI showed better identification performance for CVD than BMI, WC, and WHtR. These findings suggest that WWI may be a superior indicator of obesity, which is not limited to East Asians but generally applicable to diverse populations.

Due to the substantial differences in body composition by race, anthropometric indicators such as BMI have different cut-points for identifying obesity according to race [30]. For instance, the risk of diabetes in Chinese populations with a BMI of 26.9 kg/m² was the same as that of White populations with a BMI of 30 kg/m² [31]. Nevertheless, in the case of WWI, there were no statistically significant differences in the mean and distribution between Whites, Asians, and African Americans, supporting our current findings [32]. This may be related to the fact that WWI measures the ratio of fat and muscle mass rather than the absolute fat amount. Additionally, the Korean Frailty and Aging Cohort study showed that WWI was strongly associated with sarcopenic obesity, which is defined as the presence of high fat mass and low muscle mass combined with low physical function [33]. Therefore, elevated WWI reflects a state of excessive body fat accumulation and increased muscle mass loss. The muscle-fat imbalance results in dysregulation of adipocytokine release, inflammatory responses, endothelial dysfunction, and declined physical function, ultimately leading to the development of CVD [34–36].

The subgroup analysis demonstrated that the WWI-CVD association was more significant in participants younger than 50 years of age (*P* for interaction < 0.001). Similar to our findings, a study by Cai showed that the association between WWI and all-cause mortality was not significant at age ≥ 75 years [17]. Moreover, in the Rural Chinese Cohort Study, Li et al. found an association between WWI and hypertension in people under 60 years of age, but it disappeared in older adults (age ≥ 60 years) [37]. These results might be attributed to the different body fat distribution between older and younger individuals [38]. Moreover, subjects with obesity (BMI ≥ 30 kg/m^2^) had a higher risk for CVD than normal-weight individuals, indicating the obese population could be more vulnerable to the increase in WWI. Taken together, the risk of CVD assessed by WWI may be more advantageous for younger obese people. Besides, we found that WWI prediction ability for CVD in the Other Race subgroup was least validated, despite the fact that this measure was developed using an East Asian population. Due to the decreased sample size after stratification and the inclusion of a multi-racial population, which may lead to some potential biases, the results need to be verified in the future with larger, more specific populations.

Our study has important implications for clinical practice. First, we found a positive association between WWI and CVD in populations outside East Asia, suggesting that WWI could be a universal health index that applies to various races or ethnic groups. Besides, WWI is a better measure of obesity than BMI, WC, and WHtR, and its association with CVD is also stronger than those traditional indices. Hence, for people with high WWI levels, early assessment of target organ damage and timely intervention may reduce the risk of CVD and improve outcomes. Moreover, due to its simple calculation and economic nature, WWI can be used by medical and health institutions at all levels, especially in areas where medical resources are limited.

This study has several strengths. First of all, we used a national representative sample of the general adult population of the US from NHANES, which applied rigorous study protocols and quality controls. Secondly, we adjusted for most confounding covariates to ensure that our findings were reliable. Lastly, we used MI to maximize statistical power and minimize bias that might occur if covariates with missing data were excluded from data analyses. However, the limitations should also be noted. Due to the cross-sectional nature of NHANES, we could not obtain a causal relationship between WWI and CVD. Therefore, further longitudinal studies are needed to verify these findings. Moreover, although we have adjusted for many confounding covariates, we could not rule out any potential residual confounding, such as genetic factors and drug use. Besides, measurement errors in WC (such as the location of the waist area and individual variance in positioning the measuring tape) and CVD determined from participants’ self-reported data may lead to some potential bias. However, previous studies have demonstrated that NHANES self-reported outcomes are a valid method for determining prevalence [39]. Although WWI is a better indicator of obesity, implementing it into the current US health care system remains a challenge.

## Conclusions

Our study demonstrated that high levels of WWI were significantly associated with an increased risk of CVD in US adults, particularly in people under 50 years of age. These findings indicate that WWI may be an intervention indicator to reduce the risk of CVD in the general adult population. However, further longitudinal studies are still needed to clarify the precise causality of this relationship.

### Electronic supplementary material

Below is the link to the electronic supplementary material.

Additional File 1: Supplementary Appendix

## Data Availability

The datasets that support the findings of this study are available from the National Center for Health Statistics (https://www.cdc.gov/nchs/nhanes/index.htm) or from the corresponding author upon reasonable request.

## Abbreviations

NHANES: National Health and Nutrition Examinations Survey
CVD: Cardiovascular disease
BMI: Body mass index
WC: Waist circumference
WHtR: Waist-to-height ratio
WWI: Weight-adjusted-waist index
NCHS: National Center for Health Statistics
CDC: Centers for Disease Control and Prevention
BP: Blood pressure
HbA1c: Hemoglobin A1c
TBIL: Total bilirubin
LDL-C: Low-density lipoprotein cholesterol
HDL-C: High-density lipoprotein cholesterol
TC: Total cholesterol
TG: Triglycerides
Scr: Serum creatinine
SUA: Serum uric acid
UACR: Urinary albumin/creatinine ratio
MEC: Mobile Examination Centre
eGFR: Estimated glomerular filtration rate
CHF: Congestive heart failure
CHD: Coronary heart disease
SD: Standard deviation
OR: Odds ratios
CI: Confidence intervals
ROC: Receiver operating characteristic
MI: Multiple imputation
AUC: Area under the curve
