# Supplementary material for: Association between weight-adjusted-waist index and risk of cardiovascular diseases in United States adults: a cross-sectional study
Source: BMC Cardiovasc Disord. 2023 Sep 1;23:435. doi: 10.1186/s12872-023-03452-z (PMC10474739; doi:10.1186/s12872-023-03452-z)
Supplement: Supplementary file 1 — Additional File 1: Supplementary Appendix [file 12872_2023_3452_MOESM1_ESM.doc]

**Association between weight-adjusted-waist index and risk of cardiovascular diseases in United States adults: A cross-sectional study**

Haiyang Fang, MD ^1,#^, Feng Xie, MD ^1,#^, Kai Li, MD ^1^, Meng Li, MD, PhD ^1^,

Yanqing Wu, MD, PhD ^1,*^

**Supplementary Appendix**

**Contents**

**Supplementary Figures**

Figure S1. The flow chart of participant selection.

**Supplementary Tables**

Table S1. Multiple-imputation analysis which is based on 5 replications and the Markov-chain Monte Carlo method in the SAS multiple imputation procedure^†^.

Table S2. Adjusted odds ratios (95% CI) for association between WWI and individual CVDs.

Table S3. Pearson correlations between WWI and other obesity-related indices.

Figure S1. The flow chart of participant selection.


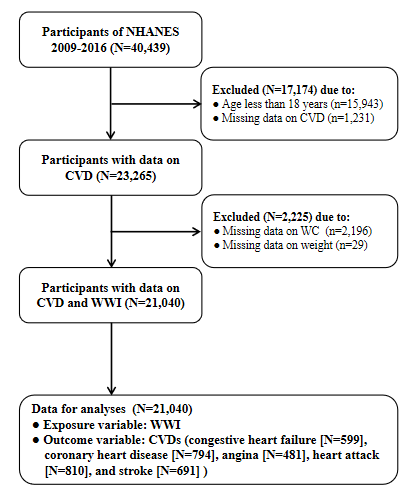


Abbreviations: NHANES, National Health and Nutrition Examination Survey; CVD, cardiovascular disease; WWI, weight-adjusted-waist index; WC, waist circumference.

Table S1. Multiple-imputation analysis which is based on 5 replications and the Markov-chain Monte Carlo method in the SAS multiple imputation procedure^†^.

| **Data** | **B** | **SE** | **OR (95%CI)** | ***P* value** |
| --- | --- | --- | --- | --- |
| Pre-imputation | 0.389 | 0.085 | 1.48 (1.25, 1.74) | <0.001 |
| Imputation 1 | 0.355 | 0.058 | 1.43 (1.27, 1.60) | <0.001 |
| Imputation 2 | 0.353 | 0.058 | 1.42 (1.27, 1.60) | <0.001 |
| Imputation 3 | 0.353 | 0.058 | 1.42 (1.27, 1.59) | <0.001 |
| Imputation 4 | 0.357 | 0.058 | 1.43 (1.28, 1.60) | <0.001 |
| Imputation 5 | 0.352 | 0.058 | 1.42 (1.27, 1.59) | <0.001 |
| Pooled estimates | 0.353 | 0.058 | 1.42 (1.27, 1.60) | <0.001 |

^†^All models adjusted the same covariates, including age, gender, race, education level, smoking, alcohol drinking, systolic blood pressure, diastolic blood pressure, estimated glomerular filtration rate, hemoglobin A1c, total bilirubin, high-density lipoprotein cholesterol, low-density lipoprotein cholesterol, total cholesterol, triglycerides, serum creatinine, serum uric acid, and urinary albumin/creatinine ratio. Abbreviations: SE, standard error; OR, odds ratio; CI, confidence interval.

Table S2. Adjusted odds ratios (95% CI) for association between WWI and individual CVDs.

| **WWI (cm/√kg)** | **Events**  **(%)** | **OR (95% CI), *P* value** | | |
| --- | --- | --- | --- | --- |
|  |  | **Model 1** | **Model 2** | **Model 3** |
| **Congestive heart failure** |  |  |  |  |
| Per 1cm/√kg increase | 599 (2.20) | 2.85 (2.53, 3.20) <0.001 | 1.97 (1.66, 2.34) <0.001 | 1.47 (1.11, 1.96) 0.008 |
| Quintile 1 (<10.3) | 26 (0.32) | Reference | Reference | Reference |
| Quintile 2 (10.3-10.8) | 56 (0.95) | 2.99 (1.74, 5.16) <0.001 | 1.84 (1.07, 3.17) 0.027 | 1.29 (0.56, 2.96) 0.549 |
| Quintile 3 (10.8-11.3) | 102 (1.92) | 6.12 (3.70, 10.12) <0.001 | 2.72 (1.63, 4.54) <0.001 | 1.56 (0.68, 3.54) 0.295 |
| Quintile 4 (11.3-11.8) | 152 (2.79) | 8.96 (5.50, 14.58) <0.001 | 3.14 (1.87, 5.26) <0.001 | 1.71 (0.70, 4.13) 0.236 |
| Quintile 5 (≥11.8) | 263 (5.98) | 19.89 (12.46, 31.76) <0.001 | 5.74 (3.43, 9.60) <0.001 | 2.67 (1.12, 6.33) 0.026 |
| *P* for trend |  | <0.001 | <0.001 | 0.002 |
| **Coronary heart disease** |  |  |  |  |
| Per 1cm/√kg increase | 794 (3.21) | 2.48 (2.24, 2.75) <0.001 | 1.65 (1.42, 1.93) <0.001 | 1.27 (1.01, 1.60) 0.045 |
| Quintile 1 (<10.3) | 30 (0.52) | Reference | Reference | Reference |
| Quintile 2 (10.3-10.8) | 83 (1.61) | 3.13 (1.80, 5.43) <0.001 | 1.69 (0.97, 2.95) 0.063 | 1.43 (0.58, 3.55) 0.431 |
| Quintile 3 (10.8-11.3) | 138 (3.12) | 6.16 (3.67, 10.33) <0.001 | 2.32 (1.36, 3.96) 0.002 | 2.12 (0.86, 5.21) 0.100 |
| Quintile 4 (11.3-11.8) | 229 (4.68) | 9.38 (5.68, 15.48) <0.001 | 2.74 (1.62, 4.66) <0.001 | 2.57 (1.06, 6.27) 0.037 |
| Quintile 5 (≥11.8) | 314 (7.32) | 15.10 (9.23, 24.69) <0.001 | 3.73 (2.20, 6.33) <0.001 | 2.26 (0.93, 5.51) 0.073 |
| *P* for trend |  | <0.001 | <0.001 | 0.014 |
| **Angina** |  |  |  |  |
| Per 1cm/√kg increase | 481 (1.96) | 2.58 (2.25, 2.97) <0.001 | 1.95 (1.61, 2.36) <0.001 | 1.44 (1.06, 1.96) 0.020 |
| Quintile 1 (<10.3) | 19 (0.38) | Reference | Reference | Reference |
| Quintile 2 (10.3-10.8) | 48 (1.00) | 2.68 (1.34, 5.36) 0.005 | 1.81 (0.90, 3.64) 0.097 | 1.51 (0.53, 4.34) 0.439 |
| Quintile 3 (10.8-11.3) | 79 (1.64) | 4.43 (2.30, 8.51) <0.001 | 2.32 (1.18, 4.59) 0.015 | 1.84 (0.62, 5.41) 0.269 |
| Quintile 4 (11.3-11.8) | 125 (2.46) | 6.69 (3.58, 12.50) <0.001 | 2.94 (1.52, 5.70) 0.001 | 2.50 (0.87, 7.16) 0.088 |
| Quintile 5 (≥11.8) | 210 (5.13) | 14.38 (7.83, 26.38) <0.001 | 5.48 (2.81, 10.70) <0.001 | 3.16 (1.05, 9.54) 0.041 |
| *P* for trend |  | <0.001 | <0.001 | 0.073 |
| **Heart attack** |  |  |  |  |
| Per 1cm/√kg increase | 810 (3.12) | 2.50 (2.28, 2.74) <0.001 | 1.89 (1.65, 2.18) <0.001 | 1.66 (1.29, 2.12) <0.001 |
| Quintile 1 (<10.3) | 29 (0.36) | Reference | Reference | Reference |
| Quintile 2 (10.3-10.8) | 72 (1.27) | 3.56 (2.03, 6.25) <0.001 | 2.29 (1.31, 4.01) 0.003 | 5.53 (2.10, 14.55) <0.001 |
| Quintile 3 (10.8-11.3) | 155 (3.40) | 9.75 (5.84, 16.28) <0.001 | 4.86 (2.88, 8.20) <0.001 | 10.53 (3.92, 28.26) <0.001 |
| Quintile 4 (11.3-11.8) | 234 (4.80) | 13.97 (8.48, 23.00) <0.001 | 5.78 (3.42, 9.79) <0.001 | 11.54 (4.16, 31.90) <0.001 |
| Quintile 5 (≥11.8) | 320 (7.03) | 20.96 (12.89, 34.10) <0.001 | 7.80 (4.65, 13.08) <0.001 | 13.68 (4.93, 38.03) <0.001 |
| *P* for trend |  | <0.001 | <0.001 | <0.001 |
| **Stroke** |  |  |  |  |
| Per 1cm/√kg increase | 691 (2.47) | 2.14 (1.92, 2.39) <0.001 | 1.40 (1.20, 1.63) <0.001 | 1.32 (1.02, 1.70) 0.036 |
| Quintile 1 (<10.3) | 41 (0.72) | Reference | Reference | Reference |
| Quintile 2 (10.3-10.8) | 81 (1.53) | 2.15 (1.33, 3.49) 0.002 | 1.38 (0.85, 2.26) 0.196 | 2.15 (0.97, 4.76) 0.059 |
| Quintile 3 (10.8-11.3) | 130 (2.32) | 3.28 (2.06, 5.22) <0.001 | 1.55 (0.97, 2.49) 0.067 | 1.78 (0.79, 4.03) 0.162 |
| Quintile 4 (11.3-11.8) | 174 (3.15) | 4.51 (2.89, 7.03) <0.001 | 1.70 (1.06, 2.73) 0.027 | 1.65 (0.72, 3.78) 0.236 |
| Quintile 5 (≥11.8) | 265 (5.43) | 7.95 (5.18, 12.21) <0.001 | 2.42 (1.52, 3.88) <0.001 | 2.19 (0.97, 4.98) 0.060 |
| *P* for trend |  | <0.001 | <0.001 | 0.083 |

Model 1: crude model;

Model 2: adjusted for age, gender and race;

Model 3: adjusted for age, gender, race, education level, smoking, alcohol drinking, systolic blood pressure, diastolic blood pressure, estimated glomerular filtration rate, hemoglobin A1c, total bilirubin, high-density lipoprotein cholesterol, low-density lipoprotein cholesterol, total cholesterol, triglycerides, serum creatinine, serum uric acid, and urinary albumin/creatinine ratio. The covariates were determined based on the matched odds ratio changed at least 10% when added to this model.

Abbreviations: WWI, weight-adjusted-waist index; CVD, cardiovascular disease; OR, odds ratio; CI, confidence interval.

Table S3. Pearson correlations between WWI and other obesity-related indices.

|  | **WWI** | **BMI** | **WC** | **WHtR** |
| --- | --- | --- | --- | --- |
| **WWI** | 1 | 0.480^*^ | 0.648^*^ | 0.793^*^ |
| **BMI** | 0.480^*^ | 1 | 0.908^*^ | 0.913^*^ |
| **WC** | 0.648^*^ | 0.908^*^ | 1 | 0.930^*^ |
| **WHtR** | 0.793^*^ | 0.913^*^ | 0.930^*^ | 1 |

*Correlation is significant at the 0.001 level. WWI, weight-adjusted-waist index; BMI, body mass index; WC, waist circumference; WHtR, waist-to-height ratio.
